# Supplementary material for: A Novel, Robust, and Portable Platform for Magnetoencephalography using Optically Pumped Magnetometers
Source: bioRxiv. 2024 Mar 11:2024.03.06.583313. Preprint. [Version 1] doi: 10.1101/2024.03.06.583313 (PMC10979878; doi:10.1101/2024.03.06.583313)
Supplement: 1 [file NIHPP2024.03.06.583313V1-supplement-1.pdf]

bioRxiv preprint doi: https://doi.org/10.1101/2024.03.06.583313; this version posted March 11, 2024. The copyright holder for this preprint (which was not certified by peer review) is the author/funder, who has granted bioRxiv a license to display the preprint in perpetuity. It is made available under aCC-BY 4.0 International license.

SUPPLEMENTARY INFORMATION

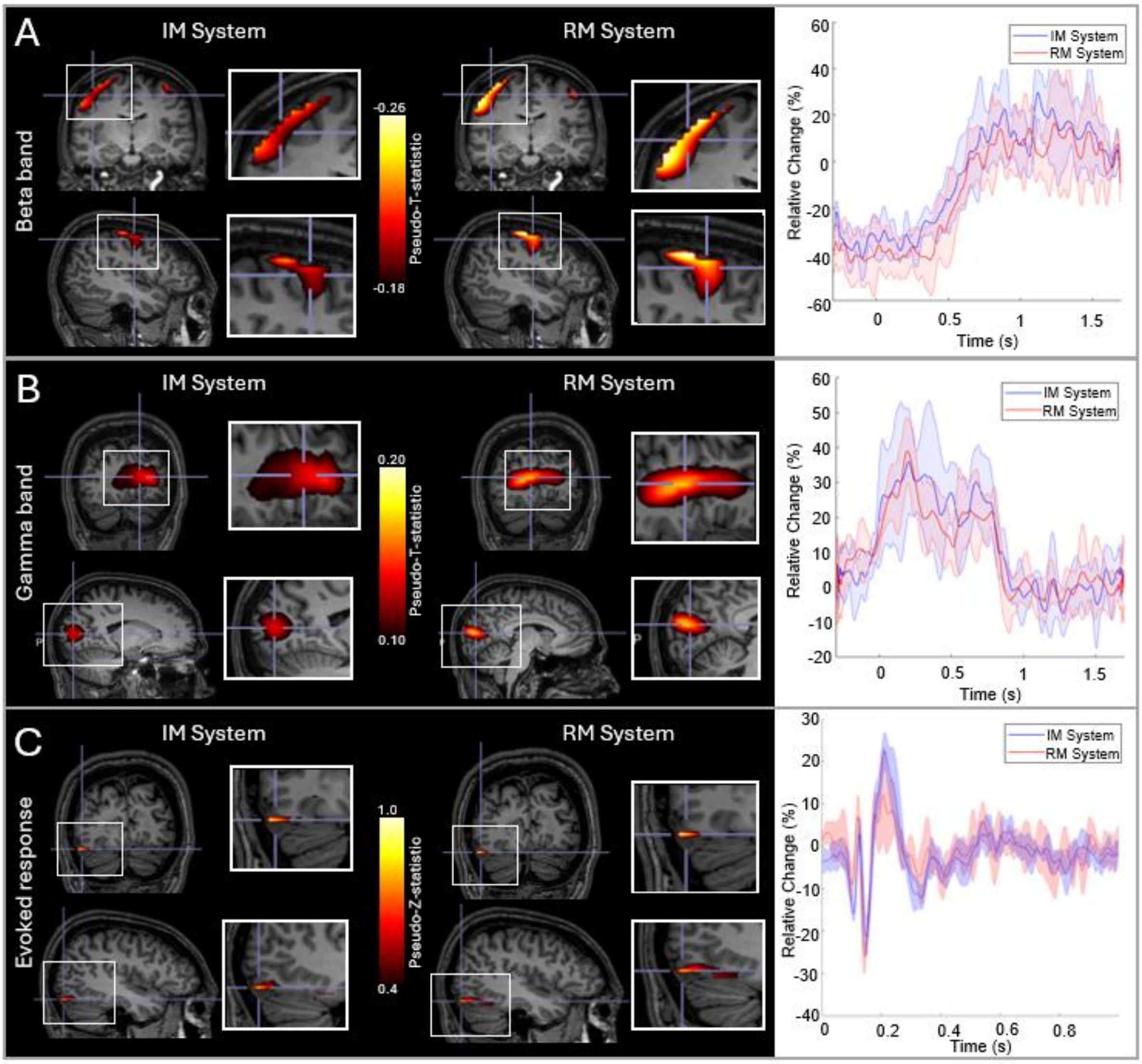

Figure S1: RM and IM system comparison for S1: *Layout equivalent to Figure 2, but results shown for Subject 1 (S1).*

bioRxiv preprint doi: https://doi.org/10.1101/2024.03.06.583313; this version posted March 11, 2024. The copyright holder for this preprint (which was not certified by peer review) is the author/funder, who has granted bioRxiv a license to display the preprint in perpetuity. It is made available under aCC-BY 4.0 International license.

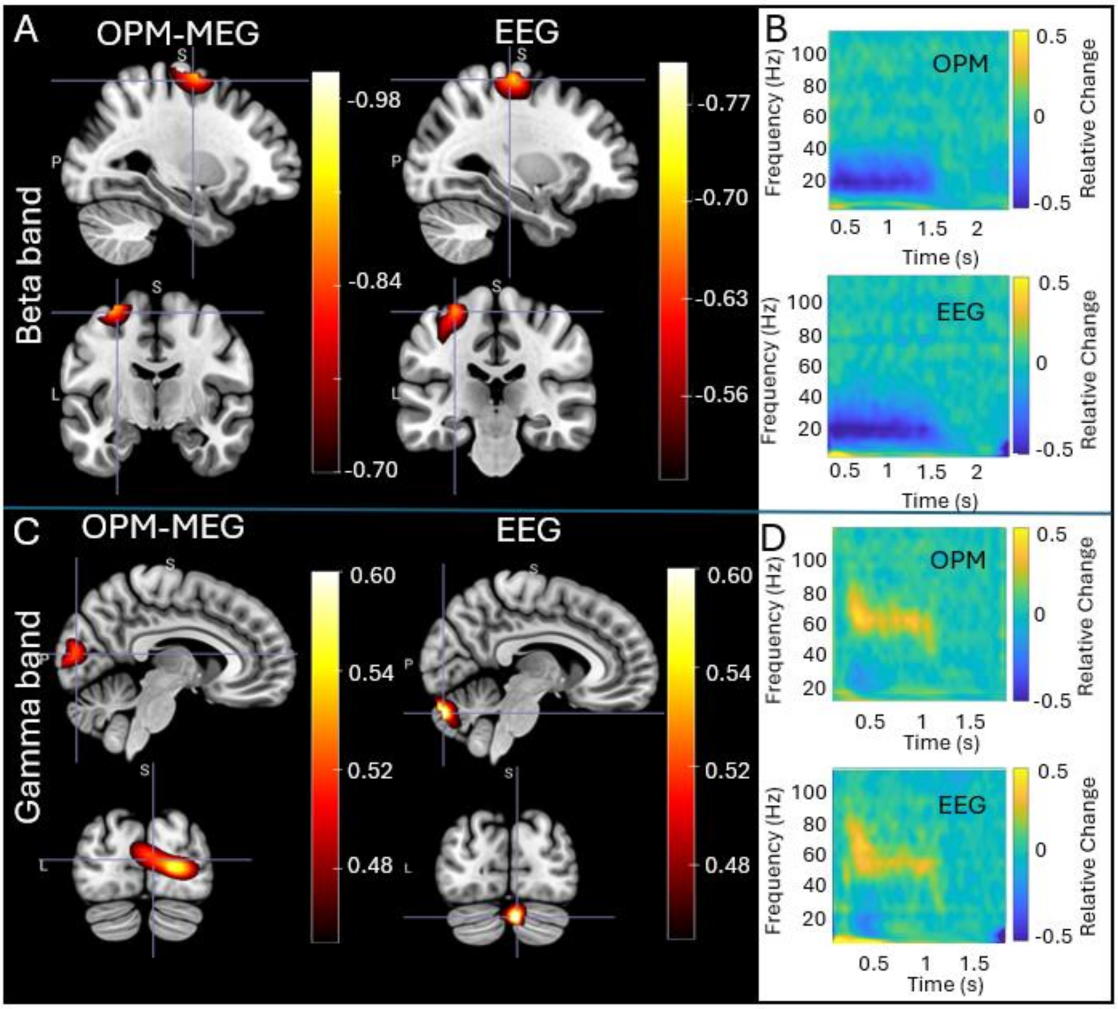

Figure S2: Concurrent OPM-MEG/EEG: *Same as Figure 6 but in the static case (i.e. no head motion).*
